# Supplementary material for: Learnable edge detectors can make deep convolutional neural networks more robust
Source: PLoS One. 2025 Sep 11;20(9):e0330299. doi: 10.1371/journal.pone.0330299 (PMC12425196; doi:10.1371/journal.pone.0330299)
Supplement: Attachment — Submitted filename: response_to_decision_letter_auresp_2.docx [file pone.0330299.s002.docx]

**Learnable Edge Detectors Can Make Deep Convolutional Neural Networks More Robust (PONE-D-25-09891R1)**

We would like to express many thanks to reviewers and associate editor for their valuable comments, which help to enhance and improve the quality of manuscript considerably. We endeavored to address all the comments and our reflections are provided below point by point. An updated version of the paper being closed is modified based on the proposed comments. Now we summarize the responses to the reviewers’ comments as follows:

**Reviewer 1**:

**Reviewer Comments:**

There is a discrepancy in the paper regarding training epochs. While it claims "The number of training epochs is set to 50000," Figure 6 shows convergence after only 60 epochs. This inconsistency needs clarification from the authors.

**Authors Responses:**

Thanks for your comments. In Figure 5 and Figure 6, all four models were trained on the CIFAR-10 dataset for 50000 epochs. To more clearly compare the training dynamics curves between BEFB-integrated models and original models, Figures 5 and 6 illustrate the changes in training loss and accuracy for the first 60 epochs. As can be seen from the figures, the BEFB-integrated models and the original models exhibit similar converging speed and accuracy. Section “Experiments--Effects of BEFB on model training” has been modified to clarify this inconsistency (marked in red).

**Reviewer Comments:**

Including only one or two papers from 2024 is insufficient for a 2025 publication. At least 10% of references should come from recent work (2024-2025). A more comprehensive analysis of recent related work is necessary.

**Authors Responses:**

Thanks for your comments. Five references from 2024 to 2025 have been added and analyzed in Section “Related Work” (marked in red).

**Reviewer 2**:

**Reviewer Comments:**

Authors addressed comments in satisfactory way and work is acceptable to be published.

**Authors Responses:**

Thanks for your efforts and time.

Finally, we would thank all reviewers again for the valuable and critical comments to make the paper more understandable and clearer.

Sincerely,

*The Authors*.
