# Supplementary material for: Learnable edge detectors can make deep convolutional neural networks more robust
Source: PLoS One. 2025 Sep 11;20(9):e0330299. doi: 10.1371/journal.pone.0330299 (PMC12425196; doi:10.1371/journal.pone.0330299)
Supplement: Attachment — Submitted filename: response to decision letter.docx [file pone.0330299.s001.docx]

**Learnable Edge Detectors Can Make Deep Convolutional Neural Networks More Robust (PONE-D-25-09891)**

We would like to express many thanks to reviewers and associate editor for their valuable comments, which help to enhance and improve the quality of manuscript considerably. We endeavored to address all the comments and our reflections are provided below point by point. An updated version of the paper being closed is modified based on the proposed comments. Now we summarize the responses to the reviewers’ comments as follows:

**Reviewer 1**:

**Reviewer Comments:**

The English in the manuscript requires significant polishing. Some transitions are too abrupt, for example: "It prompts us that, enhancing the robustness of DCNNs can be achieved by using the binary edge features which can be seen as a kind of shape features. ", the motivation needs a deeper analysis.

**Authors Responses:**

Thanks for your comments. A detailed explanation of the motivation is given at the beginning of the second paragraph of Section Introduction (marked in red).

We have proofread the manuscript and made several modifications, listed below:

[1] In the first line of Abstract and Section Introduction, “Deep convolutional neural networks (DCNN for short)” has been changed to “Deep convolutional neural networks (DCNNs)”.

[2] In the second line of Abstract, “Improving DCNN’s robustness” has been changed to “Improving DCNNs’ robustness”.

[3] In the first paragraph of Section Introduction, “which brings the potential hazards when applying DCNNs to the safety-critical applications” has been changed to “which poses potential hazards for safety-critical applications”.

[4] In the second paragraph of Section Introduction and the last paragraph of Section Proposed Approach, “learnt” has been changed to “learned”.

[5] In the second paragraph of Section Introduction, “STE technique” has been changed to “Straight-Through Estimator (STE) technique”.

[6] In the second paragraph of Section Related Work, “DCNNs are prone to be attacked” has been changed to “DCNNs are vulnerable to attacks”.

[7] In the second paragraph of Section Proposed Approach, “Inspired by the fact that shape feature is the main factor relied on by human beings to recognize objects” has been changed to “Inspired by the observation that humans primarily rely on shape features to recognize objects”.

[8] In the last paragraph of Section Proposed Approach, “Note that, the gradient of activation function of Eq. (5) is zero. To update the weights in BEFB, STE technique [14-16] is employed.” has been changed to “Note that the activation function in Eq. (5) has a zero gradient. Therefore, the STE technique [14-16] is used to update the weights in BEFB.”

[9] In the third paragraph of Section Experiments, “are on a par” has been changed to “perform comparably”.

[10] “BEFB integrated models” has been changed to “BEFB-integrated models”.

All these changes are marked in red.

**Reviewer Comments:**

The idea is similar with "DEA-Net: Single image dehazing based on detail-enhanced convolution and content-guided attention", typically, the learnable edge part. Needs proper citation.

**Authors Responses:**

Thanks for your comments. The paper “DEA-Net: Single image dehazing based on detail-enhanced convolution and content-guided attention” has been cited as Ref [15]. This paper proposed DEA-Net to perform image dehazing, part of which are four difference convolution kernels. These kernels are similar with the learnable edge detectors in our manuscript. Both can enhance the details learnt by DCNNs. The former used these enhanced details to dehaze images, and the latter leveraged these enhanced details to generate the binary shape-like features to improve robustness of DCNNs.

This paper “DEA-Net: Single image dehazing based on detail-enhanced convolution and content-guided attention” has been cited in the fourteenth line of the second paragraph in Section Introduction (marked in red).

**Reviewer Comments:**

The most recent reference cited in this work is from 2023. Given that it is now 2025, the paper would benefit from incorporating and comparing with more recent related works.

**Authors Responses:**

Thanks for your comments. The backbone models, attack methods, and robustness enhancement techniques employed in the manuscript for testing the performance of BEFB are widely used in DCNN-related research articles, e.g., ResNet34, AA, and AT. In the revised manuscript, we use WRN34-10 model, C&W attack method, BORT and DDPM robustness enhancement techniques to further test the performance of BEFB. The newly added references have been marked in red.

**Reviewer Comments:**

It seems that the proposed model not compare with SOTA models, please implement this.

**Authors Responses:**

Thanks for your comments. The performance of using the proposed BEFB to enhance robustness of DCNN is inferior than that of using SOTA robustness enhancement methods, like AT and PCL. However, when combining BEFB-integrated models with these SOTA methods, the robustness enhancement performance is better than that of combining original models with SOTA methods. The relevant experiments are conducted in Section Experiments--Combining BEFB-integrated models with SOTA robustness enhancement methods. Considering BEFB is lightweight and can be easily integrated with existing popular backbones, it depicts the superiority of BEFB. In the revised manuscript, we newly add two robustness enhancement techniques--DDPM and BORT, and the experimental results show the robustness enhancement performance of combining BEFB-integrated models with DDPM/BORT is better than that of combining original models with DDPM/BORT. For more details, please refer to Section Experiments--Combining BEFB-integrated models with SOTA robustness enhancement methods (marked in red).

**Reviewer Comments:**

If possible, please attach your code via file or opensource it.

**Authors Responses:**

Thanks for your comments. The project can be found in https://github.com/dingjin/BEFB.

**Reviewer Comments:**

The experimental setup lacks critical details. Information such as the number of training epochs, optimizer used, and learning rate settings should be explicitly provided.

**Authors Responses:**

Thanks for your comments. The number of training epochs is 50000. The optimizer used in the experiments is the SGD optimizer, with a momentum of 0.9 and a weight decay coefficient of 5e-4. The initial learning rate is set to 0.01, and the batch size for training samples is set to 128. Section Experiments-- Experimental Settings has been modified accordingly (marked in red).

**Reviewer 2**:

**Reviewer Comments:**

However, authors include only top results from 5 trainings which does not provide an appropriate statistical analysis. Authors should provide an average across those 5 trainings and standard deviation or similar metric.

**Authors Responses:**

Thanks for your comments. The values of training metrics in Table 3 have been modified to the average across 5 trainings. And the corresponding standard deviations have been provided in Table 4. Section Experiments-- Effects of BEFB on model training has been modified (marked in red).

**Reviewer Comments:**

How number of layers l and coefficient t were chosen. Why l is different for only one dataset?

**Authors Responses:**

Thanks for your comments. The number of layers *l* and coefficient *t* are chosen empirically. The Table R1 below shows the classification accuracy of BEFB-integrated models under AA attack on CIFAR-10 dataset for different *l* with *t* fixed to 0.8. The Table R2 below shows the classification accuracy of BEFB-integrated models under AA attack on CIFAR-10 dataset for different *t* with *l* fixed to 2.

Table R1 Performance comparison for different *l* with the fixed *t* = 0.8

|  | *l*=1 | *l*=2 | *l*=3 | *l*=4 |
| --- | --- | --- | --- | --- |
| VGG16-BEFB | 13.78% | **14.57%** | 14.01% | 12.72% |
| ResNet34-BEFB | 14.46% | **15.04%** | 14.73% | 14.03% |

Table R2 Performance comparison for different *t* with the fixed *l* = 2

|  | *t*=0.2 | *t*=0.4 | *t*=0.6 | *t*=0.8 | *t*=0.9 |
| --- | --- | --- | --- | --- | --- |
| VGG16-BEFB | 13.70% | 13.65% | 14.06% | **14.57%** | 13.99% |
| ResNet34-BEFB | 14.11% | 14.30% | **15.83%** | 15.04% | 14.07% |

**Reviewer Comments:**

Are both branches of the model trained? Is it training from scratch or is the backbone somehow pretrained? If backbone is also trained, how are its weight influence? Usually, some layers in CNN learn to detect edges - is this somehow changed when there is explicit training of Sobel layers?

**Authors Responses:**

Thanks for your comments. Yes. Both branches of the model are trained. Both are trained from scratch. The backbone is trained to output traditional texture features. BEFB is trained to output the binary edge features. When there is explicit training of Sobel layers, some layers in backbone we think can also learn to detect edges as common CNNs do. Fig. 8-10 demonstrate the gray features output by backbone. It can be observed edge information has been learned.

**Reviewer Comments:**

How perturbation parameters were selected?

**Authors Responses:**

Thanks for your comments. The selected perturbation parameters are commonly used in the publications on testing adversarial robustness of CNNs.

**Reviewer Comments:**

Table 4: What do bold values mean?

**Authors Responses:**

Thanks for your comments. Since the gradient of the threshold function is zero, we utilize the STE to ensure weight updates can proceed. However, when generating adversarial examples (AE) using white-box attack algorithms (such as FGSM), STE can bring obfuscated gradient effect [19-21], which means the generated AEs are not powerful enough, leading to an artificially high classification accuracy for the BEFB-integrated models. Therefore, this paper tested two alternatives to STE: the sigmoid function and zero gradient to generate AEs. The experimental results in Table 5 (Table 4 in original manuscript) show that zero gradient in most cases can allow FGSM attack to achieve lower classification accuracy of BEFB-integrated models. Consequently, we choose zero gradient for generating AEs.

**Reviewer Comments:**

Table 7: Why performance of the model on images with Gaussian noise is so much higher than the baseline one in Table 3?

**Authors Responses:**

Thanks for your comments. Adversarial examples generated with Gaussian noise do not utilize the model's gradient information, therefore the model's classification accuracy is higher. In contrast, the attack methods in Table 6 (Table 5 in original manuscript) are white-box attacks, which leverage the model's gradient information, resulting in lower classification accuracy for the model. Results in both tables show the BEFB-integrated models can have better robustness performance than the original models.

**Reviewer 3**:

**Reviewer Comments:**

The paper lacks thorough theoretical analysis of how the learnable edge detector functions. Although the authors provide visualizations of "clean vs. adversarial" edge features, the analysis of why these edge features help the model retain more shape-related cues is rather brief. A deeper discussion would help readers better understand how such structured edge features contribute to robustness against adversarial attacks.

**Authors Responses:**

Thanks for your comments. How the learnable edge detectors help improve the robustness of DCNNs is depicted in Fig. 7-10/Fig. 11-14. In these figures, the gray features are obtained from backbone branch, and the binary features are obtained from BEFB. It can be observed in Fig. 7 that, using the original VGG16 model, the difference of the extracted gray features between clean examples and adversarial examples (AE) is large, which makes AE prediction results all wrong under FGSM $\epsilon$=16 attack. While in Fig. 8, using BEFB-integrated VGG16 model, the difference of the extracted binary and gray features between clean examples and AEs is small, which keeps AE prediction results all right under FGSM $\epsilon$=16 attack. Fig. 9 and Fig. 10 add 4 more and 8 more perturbations to evaluate the performance of BEFB-integrated VGG16 model, respectively. It can be observed in both figures that, the difference of the extracted binary features between clean examples and AEs is small, demonstrating the extracted binary features from BEFB are less susceptible to the noise. Similarly, Fig. 11-14 demonstrate how the learnable edge detectors help improve the robustness of ResNet34 model. For more details, please refer to the fourth paragraph and the fifth paragraph of Section Experiments--Performance comparison between BEFB-integrated models and original models under attacks.

**Reviewer Comments:**

Adversarial attacks are more limited, and while the authors tested traditional attack methods, they did not delve into the impact of more advanced adversarial methods. Additionally, comparisons with other shape-based or multi-branch robustness enhancement methods are lacking, making it difficult to fully demonstrate the superiority of the proposed approach.

**Authors Responses:**

Thanks for your comments. In the revised manuscript, C&W attack method is included to further test the performance of the proposed BEFB. And the experimental results in Table 7 show the BEFB-integrated models perform better than original models under C&W attack on four datasets.

The performance of using the proposed BEFB to enhance robustness of DCNN is inferior than that of using SOTA robustness enhancement methods, like AT and PCL. However, when combining BEFB-integrated models with these SOTA methods, the robustness enhancement performance is better than that of combining original models with SOTA methods. The relevant experiments are conducted in Section Experiments--Combining BEFB-integrated models with SOTA robustness enhancement methods. Considering BEFB is lightweight and can be easily integrated with existing popular backbones, it depicts the superiority of BEFB.

The shape-based robustness enhancement methods require datasets of pixel-level segmentation labeling. Therefore, in the revised manuscript, we add a multi-branch robustness enhancement technique--BORT, and a SOTA robustness enhancement technique—DDPM for comparison. The experimental in Table 11 results show the robustness enhancement performance of combining BEFB-integrated model with BORT/DDPM is better than that of combining original model with BORT/DDPM. For more details, please refer to Section Experiments--Combining BEFB-integrated models with SOTA robustness enhancement methods (marked in red).

**Reviewer Comments:**

Limited innovation and model generalizability. Although the designed convolutional kernels offer a novel approach to edge detection, they are primarily limited to fixed horizontal, vertical, and diagonal edges. The effectiveness of the method on more complex edges (e.g., irregular edges) is not thoroughly investigated. Thus, the method may be mainly suitable for basic edge detection tasks, while its capability to handle more complex edge features in real-world images remains unclear. Moreover, the authors used the STE approach, however this approach is more common when dealing with similar problems. As a result, innovativeness may be lacking.

**Authors Responses:**

Thanks for your comments. The four learnable Sobel-like edge detectors are designed for detecting horizontal, vertical, positive diagonal, negative diagonal edges, which means in these directions, the detection output will be relatively strong. In other directions, these edge detectors can also work, and the detection output will be a little weaker.

When the derivative of the neural network's activation function does not exist or is zero, to the best of our knowledge, the STE is a commonly-used and effective technique to make weights can be updated. It sets the derivative to a constant value to facilitate training. In BEFB, the activation function of the threshold layer is a threshold function, and we utilize STE to enable errors can be back-propagated to the Sobel layers for weights updating. The experimental results demonstrate using STE technique, the trained BEFB-integrated models have superior performance under various attacks.

The main innovation of this paper lies in proposing BEFB to learn shape-like features to enhance the robustness of deep convolutional neural networks.

**Reviewer Comments:**

The authors should include experiments with more sophisticated adversarial strategies to further test the proposed model.

**Authors Responses:**

Thanks for your comments. In the revised manuscript, C&W attack method is included to further test the performance of the proposed BEFB. And the experimental results in Table 7 show the BEFB-integrated models perform better than original models under C&W attack on four datasets.

**Reviewer Comments:**

Comparisons with additional backbone networks should be added to validate the method's performance across different attack strategies and network architectures.

**Authors Responses:**

Thanks for your comments. In the revised manuscript, WRN-34-10 is included to further test the performance of the proposed BEFB. And the experimental results in Table 9 show the BEFB-integrated WRN-34-10 model perform better than original WRN-34-10 model under FGSM, PGD, AA, and A^3^ attacks on CIFAR-10 dataset.

**Reviewer Comments:**

It is recommended that the authors further justify the choice of this STE this method and explore whether other more effective alternatives exist.

**Authors Responses:**

Thanks for your comments. To the best of our knowledge, STE is a widely-used and effective approach to deal with non-existent or zero gradients. In BEFB, the activation function of the threshold layer is a threshold function, and we employ STE to ensure weights can be updated. In the experiments, the STE constant is set to 1. When the constant is set to other values, the classification accuracy of the trained BEFB-integrated model remains almost the same. See the table below.

Table R3 Comparison of test accuracy when training BEFB-integrated models with different STE constants on CIFAR-10 dataset

|  | 0.5 | 1 | 2 |
| --- | --- | --- | --- |
| VGG16-BEFB | 93.64% | 93.61% | 93.51% |
| ResNet34-BEFB | 93.28% | 93.26% | 93.21% |

Finally, we would thank all reviewers again for the valuable and critical comments to make the paper more understandable and clearer.

Sincerely,

*The Authors*.
